# Objectively Measured Physical Activity in Patients with Coronary Artery Disease: A Cross-Validation Study

**DOI:** 10.3390/bios11090318

**Published:** 2021-09-06

**Authors:** Tim Kambic, Nejc Šarabon, Vedran Hadžić, Mitja Lainscak

**Affiliations:** 1 Cardiac Rehabilitation Unit, Department of Research and Education, General Hospital Murska Sobota, 9000 Murska Sobota, Slovenia; 2 Faculty of Health Sciences, University of Primorska, Polje 42, 6310 Izola, Slovenia; nejc.sarabon@fvz.upr.si; 3 InnoRenew CoE, Human Health Department, Livade 6, 6310 Izola, Slovenia; 4 Laboratory for Motor Control and Motor Behavior, S2P, Science to Practice, Ltd., Tehnološki Park 19, 1000 Ljubljana, Slovenia; 5 Faculty of Sport, University of Ljubljana, 1000 Ljubljana, Slovenia; Vedran.Hadzic@fsp.uni-lj.si; 6 Division of Cardiology, General Hospital Murska Sobota, 9000 Murska Sobota, Slovenia; 7 Faculty of Medicine, University of Ljubljana, 1000 Ljubljana, Slovenia; 8 Faculty of Natural Sciences and Mathematics, University of Maribor, 2000 Maribor, Slovenia

**Keywords:** myocardial infarction, cardiovascular disease, acute coronary syndrome, sedentary behavior, motor activity, accelerometry, moderate intensity, vigorous intensity

## Abstract

Physical activity (PA) and sedentary behavior (SB) levels in healthy adults are predominately based on self-reporting measures, which generally overestimate PA but underestimate SB. Patients with coronary artery disease (CAD) eligible for cardiac rehabilitation (CR) follow an individualized program; thus, objective assessment of physical performance and regular daily activity is required. This study aimed to compare self-reported and objectively measured PA and SB in patients with CAD prior to out-patient CR. We included 91 patients with CAD and assessed their PA with an accelerometer for 8 days prior to CR, along with the short form of the international physical activity questionnaire. We found that most patients were sedentary (61%, ~8 h/day), and on average performed 63 min/day of moderate-to-vigorous-intensity physical activity (MVPA). Males performed less daily light-intensity physical activity (−5%, *p* = 0.011) and performed more MVPA (+2%, *p* = 0.002) compared to females. Maximal aerobic capacity was significantly associated with MVPA (Spearman rho = 0.483, *p* < 0.001) and MVPA > 10 min bouts (Spearman rho = 0.391, *p* < 0.001). Self-reported measures overestimated MVPA (total MVPA, +108 min/day, *p* < 0.001; MVPA > 10 min bouts, +152 min, *p* < 0.001) and underestimated SB (−174 min/day, *p* < 0.001) compared to objective measures. There was no significant correlation between methods in MVPA (Spearman rho = 0.147, *p* = 0.165)), MVPA > 10 min bouts (Spearman rho = −0.059, *p* = 576), and SB (Spearman rho = 0.139, *p* = 0.187). Quantitative analysis demonstrated the huge proportional bias for MVPA, MVPA > 10 min bouts, and SB. Our findings demonstrate that self-reported physical activity provides inaccurate estimates of MVPA and SB in patients with CAD entering the ambulatory CR. This strongly supports the more objective assessments of daily PA, preferably using an accelerometer.

## 1. Introduction

Cardiac rehabilitation (CR) has been well established as a comprehensive intervention for secondary prevention of cardiovascular diseases, with exercise training and physical activity counselling presenting two major components [1]. Patients with coronary artery disease (CAD) are advised to be moderately active at least 150 min/week or vigorously active at least 75 min/week, or a combination of both, along with a reduction in sedentary time [1]. Despite evidence and guidelines, many patients with CAD remain either sedentary (e.g., sitting, lying, napping, etc.) or with low levels of moderate to vigorous physical activity (MVPA) [2]. When compared to the healthy population, patients with CAD are less physically active and are more sedentary [2,3].

Strong evidence suggests that physical inactivity and high levels of SB are associated with an increased risk of all-cause mortality [4,5,6,7,8], cardiovascular morbidity, and mortality in healthy adults [4,6,7,9,10]. Similarly to healthy peers, low levels of PA and high levels of SB are associated with all-cause mortality and cardiovascular mortality in patients with CAD [11,12,13,14]. In addition, one study suggested that increased PA in patients with cardiovascular disease may reduce the mortality to a greater extent compared to healthy peers [13].

Most of the previous epidemiological data in healthy adults [5,6,7,8] and patients with CAD [11,12,13,14] relied on self-reported data on physical activity and sedentary behavior, which are prone to reporting bias [15]. Nevertheless, cohort observational studies using accelerometry to measure PA are emerging in healthy adults [4,9], whereas the implementation of accelerometers remains limited to only small interventional studies in patients with CAD [2,16,17,18]. In healthy adults, two recent studies have demonstrated an overlap between self-reported and objectively measured levels of MVPA per day (150–300 min vs. 30–40 min) needed to reduce the risk of mortality associated with prolonged sedentary behavior [4,19]. Similar discrepancies were observed between self-reported (using the international physical activity questionnaire) and objectively measured PA in CR [17,20]; however, this requires further investigations in patients with CAD.

To provide an accurate measure of PA in CR, this study consisted of two aims. The primary aim of the study was to validate and compare objectively measured (using three-axial accelerometry) with self-reported physical activity (using the international physical activity questionnaire short form (IPAQ-SF)) in patients with CAD. The secondary aim was to determine the physical activity and sedentary behavior characteristics of patients with CAD prior to enrollment to CR.

## 2. Materials and Methods

### 2.1. Study Design

The study was designed as a cross-sectional clinical trial. On the first ambulatory visit after acute coronary syndrome and/or percutaneous coronary intervention, patients with CAD were advised to enter the out-patient CR program. Those eventually included were asked to wear an accelerometer for 8 consecutive days within 10–14 days prior to enrolment to CR and performed a cardiopulmonary exercise test. Following the wear-time period, we collected patients’ self-reported data on physical activity and sedentary behavior using IPAQ-SF [21].

### 2.2. Participants

Patients with a stable CAD (≥1 month after acute coronary syndrome and/or percutaneous coronary intervention) were recruited from the Division of Cardiology at General Hospital Murska Sobota and were enrolled during the first ambulatory visit after hospitalization. Inclusion criteria were documented CAD and left ventricular ejection fraction ≥40%. Exclusion criteria were immobility and inability to perform any form of home and occupational activities [1]. All patients were informed about the methods, procedures, and potential risks during the study, and were asked to give their written consent prior to enrollment in the study. The study protocol was approved by the National Medical Ethics Committee (registration date: 15 June 2020; registration number: 0120-573/2019/15) and is registered with ClinicalTrials.gov (accessed on 29 October 2020, identifier: NCT04638764).

### 2.3. Data Collection and Management

#### 2.3.1. Objectively Measured Physical Activity and Sedentary Behavior

Physical activity and SB were measured with an ActiGraph wGT3X-BT accelerometer (ActiGraph, LLC, Pensacola, FL, USA). Patients were instructed on how to wear the accelerometer before attaching it to their right hip using an elastic band. The accelerometer was used during entire awake time for eight consecutive days and was removed only to avoid contact with water (e.g., showering, swimming, etc.) [22] and before going to sleep. Patients were asked about their waking and sleeping timetable during the same period.

We used ActiLife software version 6.13.4 (ActiGraph, LLC, Pensacola, FL, USA) for initialization, extraction, and analysis of data from the devices. The accelerometer was initialized for raw mode with a sampling frequency of 100 Hz at least half an hour before attaching it to the patient and was stopped on 11:00 p.m. on the eighth day of wear. The acceleration units are expressed in triaxial vector magnitude (VM) (the square root of the sum of squared activity counts) counts per min (CPM). The step count was calculated based on the manufacturer’s axial plane algorithm [22]. The raw acceleration files were converted and aggregated to 1 s epochs.agd (epoch files) using the default filter within the software. The 1 s epochs files were converted to 10 s epoch files [22] and the wear time was validated using the well-established Troiano’s algorithm [23]. The wear time was manually checked within the software, and wear time <10 min was excluded from the analysis. All deviations from usual wear time (early morning wear and/or prolonged night wear) were checked with the patients and deleted accordingly to ensure accurate wear time. Data with at least four days of 10 h wear time were included in the final analysis [22,24,25].

The three-axial VM CPM was split into different physical activity levels as follows: SB (<150 VM CPM), light physical activity (LPA) (150–2689 VM CPM), and MVPA (≥2690 VM CPM) [26,27]. The same cut-off CPM were previously used in a large cross-sectional study enrolling participants aged 40–84 years [22], which presents a similar age group as patients enrolled in CR programs [28]. We extracted the following variables for the final statistical analysis: total wear time (days), daily wear time (min/day), daily step count (steps/day), daily levels of SB (min/day and %), daily levels of LPA (min/day and %), and daily levels of MVPA (min/day and %, and min/day for MVPA bouts longer than 10 min).

#### 2.3.2. Self-Reported Physical Activity and Sedentary Behavior

Self-reported PA levels and SB were assessed using the IPAQ-SF after the end of 8 days of wear time [21]. The IPAQ short form has shown good validity and reliability in a large and diverse sample [29]. The questionnaire estimates the total amount of moderate physical activity, vigorous physical activity, walking time, and sedentary time of the past week [29]. The results were cleaned for outliers and converted to metabolic equivalents per week (MET/week) for sedentary time, walking time, moderate PA, and vigorous PA according to the recommendations (https://sites.google.com/site/theipaq/scoring-protocol, accessed on 15 July 2021). We included the following variables in final analysis: combined level and duration of MVPA (MET × min/week and min/day) and daily duration of SB (min/per day).

#### 2.3.3. Cardiopulmonary Exercise Test

Maximal aerobic capacity (VO_2_ max) was measured using an adjusted ramp protocol [30] on a Schiller ERR 911 ergometer bicycle (Schiller, Baar, Switzerland) and a Cardiovit CS-200 Excellence Ergo-Spiro system (Schiller, Baar, Switzerland). Patients performed two repetitions of spirometry, followed by 3 min rest to determine baseline blood pressure and heart rate and gas exchange. The test started with patients cycling without a workload for 3 min, followed by an increase every minute for an additional 10–20 W until exhaustion or any relevant reason to stop testing (e.g., chest pain, shortness of breath) [21]. The supervising nurse followed any potential signs or symptom-limited indications for exercise termination, as recommended by the American Heart Association [30].

### 2.4. Statistical Analysis

Descriptive variables are presented as frequencies and percentages, and numeric variables are presented as means and standard deviation for normally distributed variables, or as medians and interquartile ranges for asymmetrically distributed variables. All numeric variables were screened for normality of distribution (using Shapiro–Wilk test) and homogeneity of variances (Levene test), where appropriate. The comparison between two descriptive variables was assessed using a Chi-square test or Fisher exact test as appropriate. The gender differences were assessed using an independent samples t-test for normally distributed variables or using the Mann–Whitney test for asymmetrically distributed variables. The difference between daily recommended MVPA for patients with CAD (30 min/day of moderate PA + 15 min/day of vigorous PA = 45 min/day of MVPA) [1] and objectively measured MVPA was assessed using a one-sample t-test. The associations between objectively measured and subjectively measured PA, SB, and maximal aerobic capacity were assessed using Spearman rho correlation coefficient and interpreted as proposed previously [31]. The absolute agreement between objectively and subjectively measured PA and SB was assessed using the intraclass correlation coefficient (ICC), with the 95% confidence intervals (95% CI) for ICC [32,33]. Values of the ICC are interpreted as suggested by the recent guidelines [34]. In addition, the systematic discrepancies between objectively and subjectively measured physical activity were assessed using Bland–Altman plots [35]. Proportional bias between both measures was assessed using a one-sample t-test and using the linear model of univariate regression (independent variable: mean of both measures, dependent variable: mean differences between both measures) [35]. All analyses were performed using the IBM SPSS Software for Windows (version 25, SPSS Inc., Armonk, NY, USA) at the level of significance *p* ≤ 0.05.

## 3. Results

**Figure 1:**
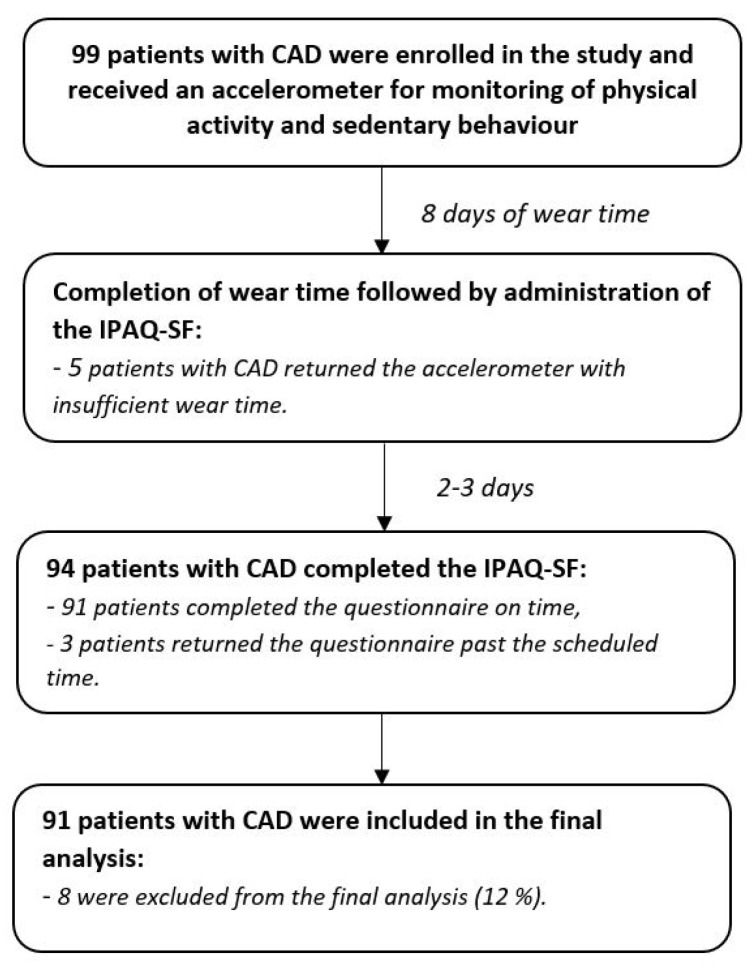
Study design and flow of the patients with CAD. CAD—coronary artery disease; the IPAQ-SF—international physical activity questionnaire short form.

Ninety-nine patients with stable CAD were enrolled in the study and the complete data of 91 patients were included in the final analysis (Figure 1).

Baseline characteristics are presented in Table 1. When compared to females, males were significantly taller (+13 cm, *p* < 0.001), heavier (+17.85 kg, *p* < 0.001) and more of them were ex-smokers prior to the event (*p* = 0.046).

Patients wore the accelerometer for more than 13 h/day, performed more than 6000 steps/day, and were predominately sedentary during the waking hours (>8 h) (Table 2). Most of the patients met the current guidelines for MVPA in CR (70%) and performed 18 min/day of MVPA (*p* < 0.001) more than recommended. Females wore the accelerometer less than males (−36 min/day, *p* = 0.012), performed less steps per day (−2308 steps/day, *p* = 0.001), and performed less MVPA (−22 min/day, *p* = 0.001; −2%, *p* = 0.002) than males. In contrast, females performed more LPA than males (+5%, *p* = 0.011). The total weekly activity score of the IPAQ-SF was classified as high in males and females, but there was no significant difference between genders.

**Figure 2:**
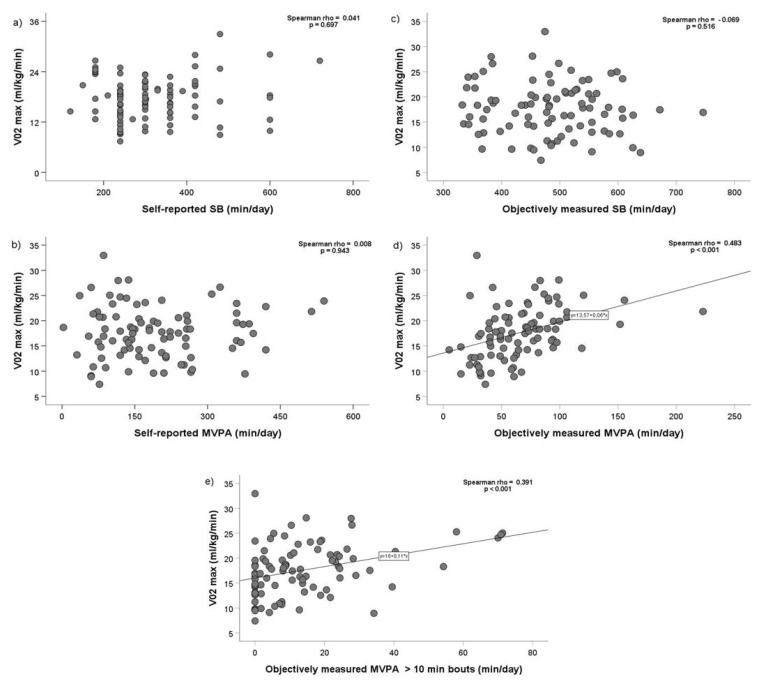
Correlations between maximal aerobic capacity, physical activity, sedentary behavior assessed by the IPAQ-SF (self-reported measure) (**a**,**b**), and accelerometry (objective measure) (**c**–**e**). VO_2_ max—maximal aerobic capacity; SB—sedentary behavior; MVPA—moderate to vigorous physical activity; IPAQ-SF—international physical activity questionnaire short form. Data are presented in scatter plots.

Figure 2 presents correlations between PA and maximal aerobic capacity. Among both methods, there were only significant and positive correlations between maximal aerobic capacity and objectively measured MVPA (Figure 2d, Spearman rho = 0.483, *p* < 0.001), and objectively measured daily MVPA > 10 min bouts (Figure 2e, Spearman rho = 0.391, *p* < 0.001).

**Figure 3:**
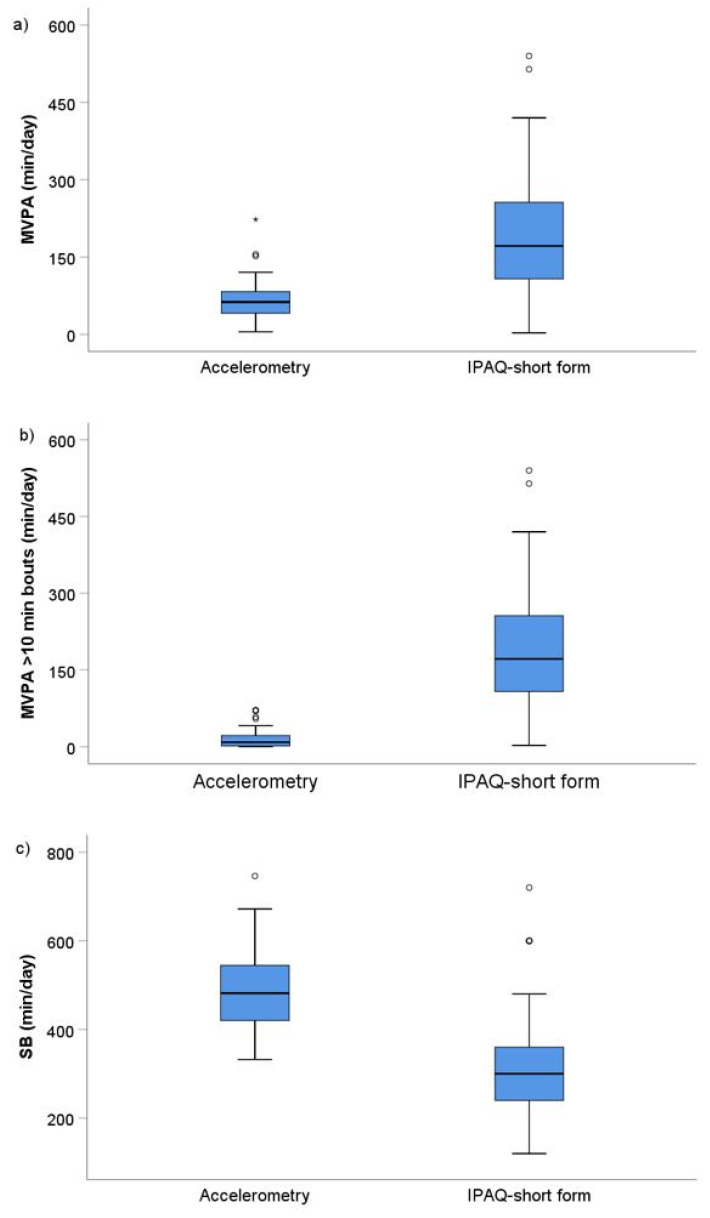
Differences between objectively and subjectively measured MVPA (**a**), daily MVPA bouts > 10 min (**b**) and SB (**c**). MVPA—moderate to vigorous physical activity; SB—sedentary behavior, IPAQ-short form—international physical activity questionnaire short form. Data are presented in boxplots. Circles and stars present outliers.

When comparing both measuring methods, there were significantly higher self-reported levels of MVPA (+108 (+39, +187) min/day; *p* < 0.001) and duration of MVPA bouts longer than 10 min (+152 (+86, +241) min/day; *p* < 0.001) and lower levels of SB (−174 (−95, −251) min/day; *p* < 0.001) compared to accelerometry data (Figure 3a–c).

Table 3 presents validation between subjectively and objectively measured PA and SB. Absolute agreement between objective and subjective measures were poor and non-significant for MVPA (ICC = 0.124, *p* = 0.088), MVPA > 10 min bouts (ICC = −0.011, *p* = 0.572), and SB (ICC = 0.090, *p* = 0.154). Similarly, there were non-significant and negligible correlations between objectively and subjectively measured MVPA (Spearman rho = 0.147, *p* = 0.165), MVPA > 10 min bouts (Spearman rho = −0.059, *p* = 0.576), and SB (Spearman rho = 0.139, *p* = 0.187).

**Figure 4:**
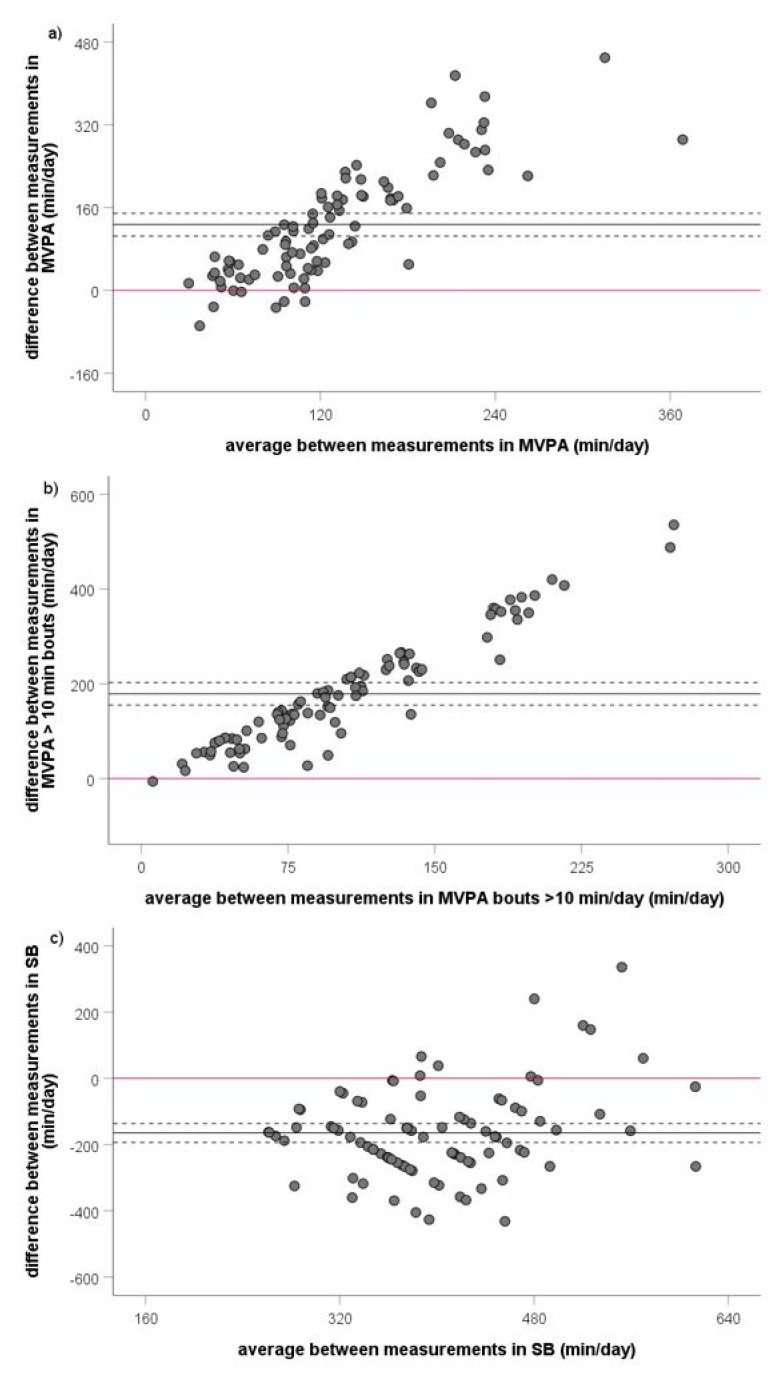
Qualitative analysis of proportional bias between objective and subjective assessment of MVPA (**a**), MVPA > 10 min bouts (**b**), and SB (**c**) using Bland–Altman plots. MVPA—moderate to vigorous physical activity; SB—sedentary behavior. Bold lines present mean value of the difference between both measures (difference = IPAQ-SF − accelerometry), dotted lines present lower and upper bound of 95% confidence interval of the difference between measures, and red bold line presents baseline.

Qualitative assessment of the systemic differences between both measures using Bland–Altman plots showed huge proportional bias for MVPA, MVPA > 10 min bouts, and SB (Figure 4a–c). In addition, quantitative analysis of proportional bias using univariate linear regression models demonstrated the significant effect of average mean of both measures on the mean difference between both measures for MVPA (Equation (1), beta value = 0.846, *p* < 0.001), MVPA > 10 min bouts (Equation (2), beta value = 0.959, *p* < 0.001), and SB (Equation (3), beta value = 0.273, *p* = 0.009). Proportional bias increased with every min/day of MVPA (error of +1.47 min/day), MVPA > 10 min bouts (error of 1.96 min/day), and SB (error of +0.49 min/day).

Mean MVPA difference between measures = −62.75 + 1.47 × mean MVPA of both measures(1)

Mean MVPA > 10 min bouts difference between measures = −23.90 + 1.96 × mean MVPA > 10 min bouts of both measures (2)

Mean SB difference between measures = −361.84 + 0.49 × mean SB of both measures(3)

## 4. Discussion

Our results demonstrate that patients with CAD are sedentary during the waking hours and their daily routine consists mostly of LPA. Male patients were more physically active than females. Our study is only the third study [17,20] to date to compare the objectively measured PA and/or SB with self-reports in patients with CAD. Self-reported PA and SB overestimated MVPA and underestimated SB. The estimated error increased by a greater extent in physically more active patients with CAD.

Physical activity presents an important component of CR programs, with partial emphasis on reducing SB and increasing MVPA [1]. Despite its importance, there are only a few studies that examined the objectively measured PA and SB prior to enrollment to CR [2,16,17,18,36,37]. When entering CR, patients with CAD were mostly sedentary (10.5–12 h/day) followed by a longer time spent in LPA (3.5 h/day). Patients with CAD rarely engaged in MVPA prior to inclusion to CR (20–65 min/day) [2,16,17,36,37]; thus, some of them failed to meet MVPA guidelines in CR [16,17,37]. This is partially in line with our findings, whereas patients performed slightly more MVPA and were less sedentary compared to some previous studies [16,36]. Furthermore, our results are also consistent with the PA levels of similarly aged healthy older adults, whereas their PA was mostly characterized as sedentary (65% of daily time) [38].

Previous studies demonstrated an overlap between males and females in SB and MVPA [2,18]. We demonstrate a significantly higher level of daily MVPA (+22 min/day, *p* = 0.001) and MVPA > 10 min bouts (+8 min/day, *p* = 0.009) in males compared with females. With the exception of daily duration of LPA and step count, our findings are similar to one of the previous studies in patients with CAD [18]. The discrepancies between studies can be explained by the level of training, as the previous study was performed in recent cardiac rehabilitation graduates; thus, females could be potentially advised to increase daily step count over the course of CR [18]. However, we obtained similar results when comparing males with females in relative daily LPA.

Maximal aerobic capacity presents a strong predictor of mortality in patients with CAD [39]; however, its associations with PA remain scarce and inconclusive. In our study, we demonstrate a positive correlation between MVPA and maximal aerobic capacity in patients prior to enrollment to CR. On the contrary, such associations were not observed in patients following CR [18]. In the latter study, the authors reported only a negative correlation between SB and maximal aerobic capacity (Spearman rho = −0.21, *p* < 0.006) [18]. This relationship is especially important in the maintenance phase after CR, wherein one study has demonstrated a beneficial role of PA monitoring on the increment in maximal aerobic capacity [40]. However, additional studies are needed to further investigate this association with emphasis on maintaining PA post CR.

Apart from a few interventional studies in CR, PA and SB were mostly assessed using self-reports in epidemiological studies in patients with CAD [3,11,12,13], whereas the benefits of MVPA and SB on mortality are usually overestimated and underestimated, respectively, compared to accelerometry data [4,19]. The recent meta-analysis in younger and older adults demonstrated the underestimation of self-reported SB compared to objectively measured SB (mean (95% CI), −105.19 min/day (−127.21 min/day, −83.17 min/day)). The authors also observed huge heterogeneity between studies, with as much as 6 h/day discrepancy in some individual studies [32]. In our study, we obtained an even larger discrepancy between both methods (−174 min/day (−251 min/day, −95 min/day), *p* < 0.001), which is similar to a recent validation study in CR (−140 min/day) [20], but in contrast to another study in patients with CAD [17]. This study did not find a difference between both methods in SB [17]. However, the authors did not report if any assistance was given to patients during the completion of the questionnaire.

Furthermore, we demonstrate an overestimation of self-reported MVPA (+108 min/day (39 min/day, 187 min/day), *p* < 0.001) and MVPA > 10 min bouts (+152 min/day (86 min/day, 241 min/day), *p* < 0.001) compared to objectively measured. Similar discrepancies were demonstrated in two previous harmonized studies of self-reported and objectively measured MVPA in healthy adults. The first study indicated that 60–75 min/day of MVPA was needed to eliminate the higher risk of death associated with SB [41], while the latter indicated only 30–40 min/day [4]. The difference between these two studies (30–45 min) can be of clinical importance in predominately sedentary patients with CAD; however, such discrepancies between studies can also be associated with the use of different levels of cut-off values for MVPA and SB [42]. For example, a different cut-off point for MVPA has provided conflicting estimates of cardiometabolic health in older adults [42].

Over the past decade, the majority of PA studies assessed MVPA using only bouts > 10 min, as this was associated with the best health outcomes [10,23]. Recently, a systematic review published by the 2018 American PA Advisory Committee showed that even daily MVPA bouts < 10 min were associated with a similar reduction in mortality [43]. This shift in the PA recommendations paradigm was also included in the recent World Health Organization PA guidelines, wherein adults are advised to accumulate as much daily MVPA as possible, regardless of the single bout duration [44]. In line with these suggestions, we validated the self-reported MVPA with both types of objectively measured MVPA and found no meaningful differences in validation outcomes. In addition, we obtained a greater association of objectively measured MVPA with maximal aerobic capacity when using total daily MVPA. Thus, it seems that daily accumulated MVPA rather than daily MVPA consisting of >10 min bouts presents a greater determinant for health-related outcomes in patients with CAD. However, due to heterogeneous protocols in previous studies in CR [2,17,18], more research implementing both methods is needed.

In line with only two available validation studies in patients with CAD [17,20], our study failed to establish the associations and absolute agreement between self-reported and objectively measured MVPA and SB. In addition, a similar pooled association in SB was observed in the recent meta-analysis, which established a low to moderate correlation between measures, with a wide 95% CI (−0.19,0.87) [32]. In generally healthy adults, the IPAQ-SF failed to reach acceptable validation compared to objectively measured PA (correlation between methods ranged from 0.09 to 0.39). The same systematic review demonstrated that the IPAQ-SF overestimated PA levels by 28% to 173% [45]. Moreover, we obtained similar proportional bias (using Bland–Altman plot) in SB and MVPA as was reported previously in patients with CAD [17] and partially in the pooled correlation data of healthy adults [32]. In addition to the available data, our study suggests that the proportional bias increased by a greater extent in physically more active patients with CAD.

In light of novel findings, we identify a few limitations of our study. Firstly, patients were advised to wear the accelerometer during waking hours, which could have been interpreted differently by each patient and might have affected the total daily wear time. However, when comparing the wear time with other studies, there was a maximal difference of 2 h [36]. In future studies, the authors should instruct patients to wear the accelerometer throughout the entire day and to record their daily waking and sleeping routine in wearing diaries. Secondly, our self-reported measure of MVPA and SB using IPAQ-SF may be influenced by the age of the patients. Although the IPAQ-SF is advised for adults aged 18–65 [29], our sample also included 34% of patients older than 65 years. Nevertheless, a similar approach was used previously in patients with CAD [17]. Thirdly, the accelerometer thresholds for LPA, MVPA, and SB were adopted from a previous study in healthy adults of similar age [22]; thus, inaccurate classification of PA and SB levels cannot be ruled out. Lastly, our sample consisted mostly of male patients, which limits the translation of our findings to female patients with CAD. Since this is common issue in CR [46], more studies should enroll female patients to provide additional evidence on their physical activity levels. 

## 5. Conclusions

Our study demonstrates that self-reporting assessment of PA overestimates MVPA and underestimates SB in patients with CAD. In addition, we also demonstrate high levels of SB prior to enrollment to CR. Therefore, objectively measured PA presents a valuable method for targeting sedentary patients with CAD, with emphasis on providing them with the optimal PA counselling to decrease SB and increase MVPA during and after CR. In conclusion, future epidemiological and/or interventional studies should use PA monitors (e.g., accelerometry data) to accurately assess the impact of PA and SB on clinical outcomes (mortality, re-hospitalization) and post-cardiac rehabilitation changes in PA and SB in patients with cardiovascular disease.

## Figures and Tables

**Table 1:** Baseline clinical and demographic characteristics.

Variable	Total Sample	Male (n = 68)	Females (n = 23)	*p*
	M (SD) or Me (Q1, Q3)	M (SD) or Me (Q1, Q3)		
**Age (years)**	62 (9)	61 (9)	63 (9)	0.304
**Anthropometrics**	**M (SD) or Me (Q1, Q3)**	**M (SD) or Me (Q1, Q3)**		***p***
Height (cm)	171.8 (8.5)	175.0 (6.5)	162.0 (5.4)	0.000
Weight (kg)	86.01 (14.89)	88.40 (80.80, 95.00)	70.55 (62.10, 83.55)	0.000
BMI (kg/m^2^)	29 (26, 32)	29 (27, 32)	28 (24, 30)	0.144
**Clinical data**	**M (SD) or Me (Q1, Q3)**	**M (SD) or Me (Q1, Q3)**		***p***
LVEF (%)	55 (45, 60)	55 (50, 60)	55 (45, 65)	0.962
Time from clinical event to inclusion to CR (months)	2.0 (1.5, 3.0)	2.0 (1.5, 3.0)	2.5 (2.0, 3.0)	0.339
**Myocardial infarction**	**f (%)**	**f (%)**	**f (%)**	***p***
NSTEMI	39 (43)	29 (43)	10 (43)	1.000
STEMI	41 (46)	31 (45)	11 (48)	
Unstable AP	10 (11)	8 (12)	2 (9)	
**Comorbidities and risk factors**	**f (%)**	**f (%)**	**f (%)**	***p***
Arterial hypertension	63 (69)	47 (69)	16 (70)	1.000
Hyperlipidemia	76 (84)	58 (85)	18 (78)	0.517
Diabetes	19 (21)	16 (24)	3 (13)	0.381
Atrial fibrillation	10 (11)	10 (15)	0 (0.00)	0.060
Thyroid disease	6 (7)	3 (4)	3 (13)	0.167
Renal disease	9 (10)	8 (12)	1 (4)	0.440
**Smoking**	**f (%)**	**f (%)**	**f (%)**	***p***
Non-smoker	28 (31)	16 (24)	12 (52)	0.046
Ex-smoker	49 (54)	40 (59)	9 (39)	
Smoker	14 (15)	12 (18)	2 (9)	
**Pharmacological therapy**	**f (%)**	**f (%)**	**f (%)**	***p***
Aspirin	89 (98)	66 (97)	23 (100)	1.000
Beta blocker	91 (100)	68 (100)	23 (100)	1.000
ACE inhibitor/ARB	90 (99)	67 (99)	23 (100)	1.000
Statin	91 (100)	68 (100)	23 (100)	1.000
Antiplatelet drug	90 (99)	67 (99)	23 (100)	1.000
Anticoagulation drug	8 (9)	7 (10)	1 (4)	0.674
Diuretic	12 (13)	10 (15)	2 (9)	0.723

M (SD): mean (standard deviation); Me (Q1, Q3): median (first quartile, third quartile); BMI: body mass index; LVEF: left ventricular ejection fraction; (N)STEMI: (non-)ST segment-elevated myocardial infarction; AP: angina pectoris; ACE: angiotensin-converting enzyme; ARB: angiotensin II receptor blockers.

**Table 2:** Objectively and subjectively measured physical activity.

Measure	Variable	Total Sample	Male	Female	∆	*p*
**Accelerometery**	Wear time (days)	8 (7, 8)	8 (7, 8)	8 (7, 8)	0	0.857
	Wear time (min/day)	798 (71)	807 (76)	771 (49)	36	0.012
	Daily step count	6422 (4878, 8426)	7183 (5479, 8871)	4875 (3791, 6572)	2308	0.001
	Daily LPA (min/day)	248 (65)	241 (61)	267 (71)	−26	0.093
	Daily MVPA (min/day)	63 (41, 83)	72 (46, 93)	50 (32, 64)	22	0.001
	Daily MVPA > 10 min bouts (min/day)	9 (2, 22)	12 (3, 24)	4 (0, 12)	8	0.009
	Daily SB (min/day)	484 (88)	493 (92)	455 (69)	38	0.073
	Daily LPA (%)	31.14 (7.66)	29.96 (7.14)	34.63 (8.21)	−4.67	0.011
	Daily MVPA (%)	7.90 (5.54, 10.30)	8.85 (5.96, 11.53)	6.59 (4.09, 7.65)	2.26	0.002
	Daily SB (%)	60.61 (9.34)	61.09 (9.50)	59.19 (8.89)	1.90	0.402
**IPAQ-SF**	Daily MVPA (min/day)	171 (104, 257)	159 (92, 257)	210 (137, 266)	−51	0.156
	Daily SB (min/day)	300 (240, 360)	300 (240, 420)	270 (240, 360)	30	0.264
	Total weekly activity score (MET, min/week)	5978 (3066, 7656)	4724 (2924, 7814)	6030 (4506, 7404)	−1306	0.293

Mean (standard deviation); median (first quartile, third quartile); objective measure: accelerometry; subjective measure: physical activity; IPAQ-SF: the international physical activity questionnaire short form; LPA: light-intensity physical activity; MVPA: moderate to vigorous physical activity; SB: sedentary behavior; MET: metabolic equivalent; ∆: difference males vs. females; d: Cohen’s d (effect size).

**Table 3:** Absolute agreement and correlation between objectively and subjectively measured MVPA and SB.

Activity	ICC	95% CI for ICC	p (ICC)	Spearman Rank rho	p (rho)
MVPA (min/day)	0.124	(−0.128,0.348)	0.088	0.147	0.165
MVPA > 10 min bouts (min/day)	−0.011	(−0.124,0.122)	0.572	−0.059	0.576
SB (min/day)	0.090	(−0.125,0.296)	0.154	0.139	0.187

MVPA-moderate to vigorous physical activity; SB-sedentary behavior; ICC-interclass correlation coefficient; 95% CI- 95% confidence interval; rho-Spearman rho correlation coefficient.

## Data Availability

The supporting data for this study are available from the corresponding author upon reasonable request.
